# Injection-Based Management of Osteoarthritis of the Knee: A Systematic Review of Guidelines

**DOI:** 10.3389/fphar.2021.661805

**Published:** 2021-04-20

**Authors:** Vito Pavone, Andrea Vescio, Matteo Turchetta, Serena Maria Chiara Giardina, Annalisa Culmone, Gianluca Testa

**Affiliations:** Section of Orthopaedics, Department of General Surgery and Medical Surgical Specialties, A.O.U. Policlinico Rodolico – San Marco, University of Catania, Catania, Italy

**Keywords:** knee osteoarthritis, injection therapy, intra-articular injections, corticosteroids, hyaluronic acid, platelet-rich plasm

## Abstract

Osteoarthritis (OA) is a leading cause of disability among older adults. Numerous pharmaceutical and nonpharmaceutical interventions have been described. Intra-articular injections are commonly the first line treatment. There are several articles, reporting the outcome of corticosteroids (CS), hyaluronic acid (HA) and platelet rich plasma (PRP). The aim of the study is to highlight the usefulness, indication and efficacy of the intra-articular injection of principal drugs. CSs have been shown to reduce the severity of pain, but care should be taken with repeated injections because of potential harm. HA reported good outcomes both for pain reduction and functional improvement. Different national societies guidelines do not recommend the PRP intra-articular injection in the management of knee OA for lack of evidence. In conclusion, the authors affirm that there is some evidence that intra-articular steroids are efficacious, but their benefit may be relatively short lived (<4 weeks). Most of the positive outcome were limited to the studies or part of the studies that considered the injection of high molecular weight as visco-supplementation, with a course of two to four injections a year.

## Introduction

Osteoarthritis (OA) is the most common form of arthritis, affecting an estimated 302 million people worldwide, and is a leading cause of disability among older adults (Neogi, 2013).

As OA spans decades of a patient’s life, patients with OA are likely to be treated with a number of different pharmaceutical and nonpharmaceutical interventions, often in combination (Pereira et al., 2011).

Here, we conduct a systematic review of international guidelines of the efficacy of intra-articular injections (IAJ) of corticosteroids (CS), hyaluronic acid (HA) and platelet rich plasma (PRP). We have the pleasure to highlight the usefulness of bringing together a summary of international guidelines in one place, being more than one guideline on these important topics. It should help clinicians to review and appraise published guidelines systematically, and aid evidence-based clinical decision-making.

Currently, there is no critical appraisal of international guidelines that are synthetized, graded and comprehensively presented for injection-based therapy of knee OA.

The aim of the study is to highlight the usefulness, indication and efficacy of the intra-articular injection of principal drugs. Therefore, a systematic critical appraisal of international guidelines was undertaken to comprehensively present the evidence-based recommendation on injection-based therapy of knee OA.

## Methods

### Searching and Guidelines Selections

The MEDLINE, Cochrane Library, SPORTDiscus with Full Text, ScienceDirect, Scopus, CINAHL, Google Scholar and PEDro databases were investigated to detect all most recent guidelines, protocols, and recommendations for the management or treatment of knee OA. In addition, Internet searches of all relevant arthritis organizations were undertaken. Guidelines that included intra-articular steroid injection, hyaluronic acid injection and platelet rich plasma injection, against placebo for osteoarthritis of the knee were included (Table 1). Six guidelines were identified for evaluation, selecting just those considered “recommended” after the application of the AGREE II (AGREE, 2009). One was excluded (Kolasinski et al., 2020), because the recommendation was not clear for the AGREE II appraisal guidelines tool. Due to high clinical impact of American College of Rheumatology/Arthritis Foundation (ACR) guidelines, the society recommendations were included as “warming”.

**TABLE 1:** Intra-articular steroid injection, hyaluronic acid injection and platelet rich plasma injection recommendations studies.

Organization (Year)	Scope and purpose	Stakeholder involvement	Rigor of development	Clarity of presentation	Applicability	Editorial indipendence	Overall quality assessment rating	Effectively addressed or not
ACR (2019)	89	56	79	83	4	8	Recommended with modifications	<60%
							Recommendations need to be more specific	
OARSI (2019)	89	56	94	89	21	58	Recommended	>60%
AAOS (2013)	94	67	96	100	0	58	Recommended	>60%
EULAR (2020)	78	33	60	78	21	8	Recommended	>60%
RACGP (2018)	100	61	88	83	29	58	Recommended	>60%

ACR, American College of Rheumatology; OARSI, Osteoarthritis Research Society International; AAOS, American Academy of Orthopedic Surgeons; EULAR, European League Against Rheumatism; RACGP, Royal Australian College of General Practitioners.

The OA guidelines used from evidence-based investigation, consensus, and/or expert opinion were considered eligible and included. No restrictions on severity or site of OA, sex, or age were applied.

### Quality Appraisal and Data Extraction

Appraisal of Guidelines for REsearch and Evaluation II (AGREE II) instrument was chosen to assess the quality of all guidelines. The assessment of each was study was performed independently. Sixty percent was chosen as the adequate coverage value for domains (5,6). For each guideline recommendation, the related involvements were ranked on an individual weighting scale from +3 to −1. Recommendations were rated and collected according to median score into strongly recommended, recommended, recommended with caution, and unsupported. If one intervention is recommended by 4 guidelines with rates of 2, 2, 2, 2, resulting in a median score of 2 and grading of recommended (Table 2).

**TABLE 2:** Recommendations graded and grouped based on their median score.

	CS injection	HA injection	PRP injection
OARSI	1	1	-1
AAOS	0	-1	-1
EULAR	2	2	-1
RACGP	1	-1	-1
Median	1	0,25	-1
ACR	2	1	-2

OARSI, Osteoarthritis Research Society International; AAOS, American Academy of Orthopedic Surgeons; EULAR, European League Against Rheumatism; RACGP, Royal Australian College of General Practitioners; ACR, American College of Rheumatology/Arthritis Foundation; CS, Corticosteroids; HA, Hyaluronic acid; PRP, Platelet-rich plasma; 2, strongly recommended; 1, recommended; 0, no evidence for recommendation; -1, recommendation against; -2, strong recommendation against)

### The AGREE II

The critical assessment of the guidelines was performed according to Appraisal of Guidelines for REsearch and Evaluation II (AGREE II). AGREE II is a high construct validity quality appraisal scale. It composed of 23 items organized into 6 domains: scope and purpose (3 items), stakeholder involvement (3 items), rigor of development (8 items), clarity of presentation (3 items), applicability (4 items), and editorial independence (2 items). Each item is ranked between strongly agree and strongly disagree. Percentage score was calculated for each domain. A domain was evaluated effective addressed and recommended if its score was 60%.

## Results

### Corticosteroid Injections

In 1953, Dr. Hollander and collegues from University of Pennsylvania were first authors who described interarticular corticosteroid injections for patients with rheumatoid arthritis. Subsequently, have become a commonly utilized technique of managing pain in KOA affected patients as well, and previous reviews on the topic has generally found them to be an effective treatment. IAJ for knee OA have been demonstrated to decrease the pain gravity (McCrum, 2016).

The 2019 Osteoarthritis Research Society International (OARSI) international guidelines conditionally recommended (recommended with caution) the use of intra-articular corticosteroids knee OA affected patients in all groups. A Good Clinical Practice Statement utilizing to intra-articular (IA) treatments for all comorbidity subgroups was added, noting that intra-articular corticosteroid (IACS) may provide short term pain relief (Bannuru et al., 2019).

In the latest international guidelines, the American Academy of Orthopedic Surgeons (AAOS) (Brown, 2013) does not currently have recommendations for or against the use (unsupported) of IACS injection of the knee and advises that practitioners should be alert for emerging evidence that clarifies or helps determine the balance between benefits and potential harm: “We are unable to recommend for or against the use of intraarticular (IA) corticosteroids for patients with symptomatic osteoarthritis of the knee”.

2020 European League Against Rheumatism (EULAR) recommendations for the management of knee osteoarthritis claims that “Intra-articular injection of long acting steroid is indicated for acute exacerbation of knee pain, especially if accompanied by effusion” (recommended) (Pendleton et al., 2000).

The Royal Australian College of General Practitioners (RACGP) in its Guidelines for the management of knee and hip osteoarthritis published in 2018 assert that “Corticosteroid injections could be offered for short-term symptom relief for some people with knee OA, but care should be taken with repeated injections because of potential harm”, giving to the physician a conditional recommendation for the intervention (recommended with caution) (The Royal Australia College of General Practitioners, 2018).

American College of Rheumatology/Arthritis Foundation (ACR) in the society guideline highlighted that “The Intraarticular glucocorticoid injections are strongly recommended for patients with knee and/or hip OA”, due to the good short-term outcome, even if the main disadvantage of cartilage loss is caused by specific steroid preparations with a certain frequency of injections (Kolasinski et al., 2020).

### Hyaluronic Acid Injections

The knee OA IAHA clinical benefit on may depend on two mechanisms: joint mechanical viscosupplementation (permitting lubrication and shock absorption), and the re-creation of joint homeostasis by encouraging endogenic HA production, which endures long after the exogenous injection has left the joint (Maheu et al., 2016).

The latest Osteoarthritis Research Society International (OARSI) international guidelines published in 2019 conditionally recommended (recommended with caution) hyaluronic acid injections in individuals with knee OA in all groups. A Good Clinical Practice Statement applying to intra-articular (IA) treatments for all comorbidity subgroups was added, noting that intra-articular hyaluronic acid (IAHA) may have beneficial effects on pain at and beyond 12 weeks of treatment (Bannuru et al., 2019).

In the 2013 international guidelines the American Academy of Orthopedic Surgeons doesn’t recommend (not recommended] using hyaluronic acid for individuals affected by symptomatic knee OA: “Meta-analysis in meaningfully important difference (MID) units showed that the over effect was less than 0.5 MID units, indicating a low likelihood that an appreciable number of patients achieved clinically important benefits in the outcomes. Although meta-analyses of WOMAC pain, function, and stiffness subscales scores all found statistically significant treatment effects, none of the improvements met the minimum clinically important improvement thresholds. When we differentiated high- vs. low- molecular weight viscosupplementation, our analyses did show that most of the statistically significant outcomes were associated with high-molecular cross linked hyaluronic acid but when compared to mid-range molecular weight, statistical significance was not maintained” (Brown, 2013).

2020 EULAR recommendations for the management of knee osteoarthritis support the use (recommended) of hyaluronic acid injections: “there is evidence to support the efficacy of hyaluronic acid in the management of knee OA both for pain reduction and functional improvement” (Pendleton et al., 2000).

The Royal Australian College of General Practitioners in its Guidelines for the management of knee and hip osteoarthritis published in 2018 states that “We suggest not offering viscosupplementation injection for people with knee OA”, giving to the practitioners a conditional recommendation against the procedure (not recommended) (The Royal Australia College of General Practitioners, 2018).

In 2020, ACR recommended a limited use of IAHA, especially for glucocorticoid injections or in cases of interventions fail in reducing local joint symptoms. The restricted IAHA benefit findings, when other alternatives have been exhausted or failed to provide satisfactory benefit, is the main reason of conditional recommendation against (Kolasinski et al., 2020).

### Platelet Rich Plasma Injections

The 2019 Osteoarthritis Research Society International (OARSI) international guidelines strongly recommended against Platelet-rich plasma treatment in patients with knee OA: “there is concern regarding the heterogeneity and lack of standardization in available preparations of platelet-rich plasma, as well as techniques used, making it difficult to identify exactly what is being injected” (not recommended) (Bannuru et al., 2019).

Also in the latest (2013) international guidelines of the American Academy of Orthopedic Surgeons, PRP is cited among the treatments that are not recommended for knee OA (not recommended) (Brown, 2013).

PRP usage in the management of knee OA is taken into account in 2020 EULAR recommendations among the treatments that are not recommended for lack of evidence (not recommended) (Pendleton et al., 2000).

The Royal Australian College of General Practitioners in its Guidelines for the management of knee and hip osteoarthritis published in 2018 declare that they recommend against the use of PRP injection for people with knee OA due to a lack of high-quality evidence (not recommended) (The Royal Australia College of General Practitioners, 2018).

This review is the first critical appraisal of guidelines for the injection-based therapy of knee OA. Of the 5 guidelines that were identified, 1 was excluded.

The heterogeneity and lack of standardization in available preparations of platelet-rich plasma, as well as techniques used, making it difficult to identify exactly what is being injected are the principal reason of ACR strong recommendation against PRP injection in KOA (Kolasinski et al., 2020).

## Discussion

An overall recommendation was provided according to the specific interventions across guidelines median calculation. These are presented as strongly recommended, recommended, recommended with caution, unsupported, and not recommended. None of the procedures were strongly recommended and recommended. Corticosteroid injection was recommended with caution. The use of hyaluronic injection was considered unsupported. The PRP injection was not recommended.

We observed a great discordance through guidelines on the recommendations for the use of corticosteroid injection and hyaluronic acid injection; the former was recommended with caution in one guideline, unsupported in one guideline, recommended in one guideline and recommended with caution in one guideline, the latter was recommended with caution in one guideline, explicitly not recommended in two guidelines and recommended in one guideline.

Instead, we noticed agreement in all retrieved guidelines PRP injections utilize in knee osteoarthritis; it was not recommended due to a lack of evidence.

Giving a closer look to the recommendations of the guidelines on the use of corticosteroid injection and analysing the studies considered, five mild quality trials that compared intraarticular corticosteroids to placebo (Gaffney et al., 1995; Jones and Doherty, 1996; Ravaud et al., 1999; Raynauld et al., 2003; Chao et al., 2010; Maheu et al., 2016) were found. All the studies were considered valid for hypothesis, blinding, treatment integrity or measurement domains. All five studies were flawed in the cohort assignment domain, and one study were flawed in the cohort comparation and researcher bias domains (Chao et al., 2010). These studies assessed relief of pain for a 4 weeks period. Four of these studies didn’t find corticosteroid to be superior to placebo at any time. One study included 98 patients demonstrated after 1 week a considerable variation between intra-articular steroid and placebo (Ravaud et al., 1999).

Only one study found intra-articular corticosteroids to be superior to placebo on Western Ontario and Mc Master University (WOMAC) total subscale scores at 4 weeks (Chao et al., 2010).

In conclusion, the authors affirm that there are some proofs that intra-articular steroids are successful, but their advantage may be quite short lived (<4 weeks). However additional findings are necessary to consider the use of corticosteroid injection as a standard therapy. We strongly encourage the develop of study protocol and randomized clinical trials.

Regarding the recommendations on the use of hyaluronic acid injection, on reviewing the 10 studies (Dougados et al., 1993; Puhl et al., 1993; Carrabba et al., 1995; Corrado, et al., 1995; Karlsson et al., 2002; Day et al., 2004; Lee et al., 2006; Jüni et al., 2007; Lundsgaard et al., 2008; Raman et al., 2008; Huang et al., 2011; Rutjes et al., 2011) taken into account in the guidelines that compared HA injection vs. placebo, we found that there is some proofs to sustain the utilization of high molecular weight viscosupplementation (Rutjes et al., 2011), while for other agents the evidence is weak or absent. Even if our analysis revealed that benefit was limited to the experiments with higher risk of bias: when restricted to experiments with low risk of bias, meta-analysis has demonstrated that the impact size of hyaluronic acid injections compared to saline injections approaches zero (Rutjes et al., 2011). Most of the positive outcome were limited to the studies or part of the studies that considered the injection of high molecular weight viscosupplementation and a course of two to four injections a year (Carrabba et al., 1995; Lee et al., 2006).

In summary, there is some findings to sustain the efficiency of hyaluronic acid in the managing of knee OA for pain decrease, but quality of the evidence is still inadequate and the positive outcome are limited to high molecular weight hyaluronic acid.

These projections even more underscore the need to know and understand the guidelines for injection-based treatment, since that pain associated with OA often leads to inactivity and loss of mobility, resulting in deconditioning, weight gain, loss of independence, and decreased quality of life (Loew et al., 2012); while a good injection-based management of OA of the knee can foster more physical activity and exercise that can modify several biohumoral indicators having a positive effect on the health of elderly (Fossati et al., 2020). Furthermore, even if joint substitution is an efficient intervention to relieve pain and enhance quality of life for those with advanced OA. However, despite a increasing amount of joint replacements undertaken each year, many people are still included on a waiting list often for a significant time and a large number of patients affected with knee OA do not like to undergo surgery for associated comorbidity (Williams et al., 1997; Ackerman et al., 2011).

### Study Limitation

Only guidelines published in English were reviewed, leading to a potential publication bias.

The objective of this appraisal was to examine the accessible guidelines and offer the treatment recommendations for the injection-based management of OA in a format that was helpful to the physician. EULAR Guidelines are collection and adapted/translated the data local National Societies version summery. The guideline reflect the main clinical practice in Europe.

## Conclusion

In conclusion, there are some proofs that intra-articular steroids are efficacious, but their advantages may be relatively short lived (<4 weeks). Most of the positive outcome were limited to the studies or part of the studies that considered the injection of high molecular weight as visco-supplementation, with a course of two to four injections a year. In this critical appraisal we assessed the quality of the guidelines, synthesized, rated, and meticulously offered all the significant recommendations for the injection-based treatment of OA of the knee. It is expected that this will advise health care providers on the best findings interventions accessible for the injection-based therapies of the knee OA.
